# Quantitative Approach for Incorporating Methylmercury Risks and Omega-3 Fatty Acid Benefits in Developing Species-Specific Fish Consumption Advice

**DOI:** 10.1289/ehp.11368

**Published:** 2008-09-03

**Authors:** Gary L. Ginsberg, Brian F. Toal

**Affiliations:** Connecticut Department of Public Health, Hartford, Connecticut, USA

**Keywords:** cardiovascular risk, fish advisory, methylmercury, neurodevelopment, omega-3 fatty acids, risk/benefit

## Abstract

**Background:**

Despite general agreement about the toxicity of methylmercury (MeHg), fish consumption advice remains controversial. Concerns have been raised that negative messages will steer people away from fish and omega-3 fatty acid (FA) benefits. One approach is to provide advice for individual species that highlights beneficial fish while cautioning against riskier fish.

**Objectives:**

Our goal in this study was to develop a method to quantitatively analyze the net risk/benefit of individual fish species based on their MeHg and omega-3 FA content.

**Methods:**

We identified dose–response relationships for MeHg and omega-3 FA effects on coronary heart disease (CHD) and neurodevelopment. We used the MeHg and omega-3 FA content of 16 commonly consumed species to calculate the net risk/benefit for each species.

**Results:**

Estimated omega-3 FA benefits outweigh MeHg risks for some species (e.g., farmed salmon, herring, trout); however, the opposite was true for others (swordfish, shark). Other species were associated with a small net benefit (e.g., flounder, canned light tuna) or a small net risk (e.g., canned white tuna, halibut). These results were used to place fish into one of four meal frequency categories, with the advice tentative because of limitations in the underlying dose–response information. Separate advice appears warranted for the neurodevelopmental risk group versus the cardiovascular risk group because we found a greater net benefit from fish consumption for the cardiovascular risk group.

**Conclusions:**

This research illustrates a framework for risk/benefit analysis that can be used to develop categories of consumption advice ranging from “do not eat” to “unlimited,” with the caveat that unlimited may need to be tempered for certain fish (e.g., farm-raised salmon) because of other contaminants and end points (e.g., cancer risk). Uncertainties exist in the underlying dose–response relationships, pointing in particular to the need for more research on the adverse effects of MeHg on cardiovascular end points.

A decade ago, the landmark studies from the Seychelles (Davidson et al. 1998) and Faroe islands (Grandjean et al. 1997) were unfolding and a debate was raging over how much risk is associated with methylmercury (MeHg) in fish. Both the Seychelles and Faroe studies involved populations that have a high per capita consumption of fish and MeHg body burdens generally higher than in the United States (Davidson et al. 1998; Grandjean et al. 1997). The Seychelles study showed no evidence of harm, whereas the Faroe study, at similar MeHg exposure levels, showed significant neurodevelopmental deficits at birth and into the early school years (Axelrad et al. 2007). Interpretation of these studies by the U.S. Environmental Protection Agency (EPA) and Agency for Toxic Substances and Disease Registry (ATSDR) differed, creating confusion in federal and state government over how to set fish consumption advice (ATSDR 1999; U.S. EPA 2001). A National Academy of Sciences report [National Research Council (NRC) 2000] helped resolve the debate by concluding that MeHg in fish is an important public health risk and developed a dose–response analysis for neurodevelopmental effects that was subsequently used by the U.S. EPA to derive the reference dose (RfD) (U.S. EPA 2001). The Seychelles study, although still overall a negative (without effects) study, recently found some evidence suggestive of a latent MeHg effect (Davidson et al. 2006). An ongoing study of a birth cohort in Massachussetts shows an association of MeHg exposure with neurodevelopmental effects at lower levels of exposure than in prior studies (Oken et al. 2005, 2008).

One might assume that the controversy is over. The issue has been through the National Academy of Sciences, and public health officials now have an RfD on the U.S. EPA’s Integrated Risk Information System (IRIS) database (U.S. EPA 2001) that can be used to set fish consumption limits. Why, then, is the subject of fish consumption still as debatable now as it was a decade ago? The answer is that the nutrients in fish, especially the fish oil omega-3 fatty acids (FAs) eicosapentaenoic acid (EPA; C_20:5_ n-3) and docosa hexaenoic acid (DHA; C_22:6_ n-3) have been increasingly identified as having public health benefits. This leads to the concern that avoiding fish because of contaminants will eliminate the benefits from fish consumption, a concern heightened by the fact that the most abundant natural source of EPA and DHA is fish (Racine and Deckelbaum 2007). Balancing the risks and benefits of fish consumption has become an increasingly important goal of fish consumption advisories. However, recent messages in the media that emphasize fish benefits have created confusion about the need for caution (Hobson 2006). In one case, an advocacy group recommended that pregnant women exceed federal fish consumption guidelines, but that group has subsequently been found to have dubious funding sources (Couzin 2007). On the other hand, warnings about MeHg levels in fish can provide overly negative messages that cause women to completely avoid fish (Cohen et al. 2005a; Oken et al. 2003).

In this article we quantitatively address key aspects of the fish risk/benefit issue by analyzing the health trade-offs for individual fish species. Although MeHg and omega-3 FA are both present in fish, species can be distinguished based on the relative proportion of these constituents (Mahaffey et al. 2007; Stern 2007). The present analysis provides a quantitative approach for identifying which fish are most beneficial for neurodevelopmental and cardiovascular outcomes. Our focus is on the potential utility of the approach rather than the exact results obtained to date, because uncertainties in the underlying dose response make the conclusions tentative. Although showing possible directions for species-specific advisories, the analysis points to key research areas for improving risk/benefit analysis for fish consumption. The demonstrated approach may serve as a model for analyzing fish species, contaminants, and end points not included in the present analysis.

## Evidence of fish consumption effects on neurologic and cardiovascular outcomes

The ingestion of fish or fish oils has been associated with an array of health benefits, including improvement of blood lipid profiles, decreased risk of heart disease, and lowered blood pressure [Institute of Medicine (IOM) 2006; Mozaffarian and Rimm 2006], improvement in rheumatoid arthritis (Kremer 2000), enhanced eye and brain development in early life (Fleith and Clandinin 2005), prevention of macular degeneration (SanGiovanni et al. 2007), lower risk of colitis (Hudert et al. 2006) and type 2 diabetes (Barre 2007), and improvement in neurologic and psychological disorders such as depression, schizophrenia, and Parkinson disease (Calon and Cole 2007). Diets rich in omega-3 FA increase the ratio of omega-3 to omega-6 (primarily from vegetable sources) in cell membranes. This, as well as a host of related effects on lipid chemistry, leads to a generalized antioxidant, anti-inflammatory effect that has documented benefits in neural tissues, vascular endothelium, and cardiac muscle (antiarrhythmic effect) (Connor 2000; Farooqui et al. 2007; Massaro et al. 2006; Mozaffarian and Rimm 2006; von Schacky 2006).

The purported benefits of fish oil omega-3 FA are perhaps best documented for cardiovascular end points and enhanced brain development. It is noteworthy that MeHg also has toxic effects in these areas. Therefore, the present analysis focuses on fish consumption risks and benefits on these end points. The following sections provide a brief review of pertinent literature in these areas as background for our quantitative species-specific risk/benefit analysis.

## Fish and omega-3 FA effects on cardiovascular end points

Recent reviews of the cardiovascular benefits from fish and fish oils have focused on mortality from coronary heart disease (CHD; IOM 2006; Mozaffarian and Rimm 2006; von Schacky 2007). Evidence from a combination of 20 different prospective cohort studies and clinical trials has shown a consistent decline in CHD mortality with increasing omega-3 FA intake (EPA + DHA) with an apparent saturation of this benefit at intakes > 250 mg/day (Mozaffarian and Rimm 2006). Below an ingestion rate of 250 mg/day, there was a 14.6% decrease in CHD mortality per 100 mg/day omega-3 FA ingested (95% confidence interval, 8–21% reduction). CHD benefits were strongest for oily fish such as salmon, herring, and sardines relative to leaner fish (cod, catfish, halibut). Although the weight of evidence supports a cardiovascular health benefit from fish oils, not all analyses have found this to be the case (Hooper et al. 2006).

The meaning of the saturation of benefit in the Mozaffarian and Rimm (2006) pooled analysis is unclear because it contains studies in which omega-3 FA ingestion was from fish in some cases and from omega-3 FA supplements in others, with this not clearly segregated in their analysis. Saturation of benefit above 250 mg omega-3 FA intake per day may not be an actual plateau, because as fish ingestion increases, so does the intake of MeHg. The toxicity of MeHg on the same cardiovascular end point may cause a net leveling off of the benefit. Separate evaluation of omega-3 FA supplementation studies is needed to refine the analysis, but in general, there are fewer of these studies and they were not designed to evaluate dose response (Konig et al. 2005). In one particular case, supplementation of the diet of Japanese adults who have cardiovascular disease with 1.8 g/day EPA yielded a measureable benefit on CHD mortality (von Schacky 2007). Given the high level of fish consumption and therefore the high background of omega-3 FA intake in this population, the added benefit from supplemental fish oil suggests that the benefit does not saturate. If this is true, the apparent saturation reported by Mozaffarian and Rimm (2006) may in fact reflect the counterbalancing effect of MeHg. Additional research in this area is needed.

Fish oil may also have benefits on a variety of other cardiovascular end points, including decreases in nonfatal myocardial infarction (MI), ischemic stroke, atrial fibrillation, atherosclerosis, and congestive heart disease (IOM 2006; Mozaffarian and Rimm 2006; von Schacky 2007). However, the evidence in these cases is limited and not currently suitable for a risk/benefit assessment of fish consumption.

## MeHg effects on cardiovascular end points

MeHg is a risk factor for cardiovascular disease through a variety of mechanisms potentially involving pro-oxidant effects via the generation of radical species and the inactivation of cellular antioxidant systems such as glutathione peroxidase and catalase (Guallar et al. 2002). There is evidence for lipid peroxidation and elevations of oxidized low-density lipoprotein in association with MeHg exposure (Andersen and Andersen 1993; Salonen et al. 1995). Mechanistic studies indicate that MeHg can exert toxic effects on the vascular endothelium by depletion of sulfhydryls, increased oxidative stress, and activation of phospholipases (Hagele et al. 2007; Mazerik et al. 2007). Oral dosing of rats with MeHg at a daily rate of 0.5 mg/kg for 9 months yielded a persistent pressor effect (Wakita 1987), whereas inorganic mercury has caused a variety of adverse effects on cardiovascular function, including increased blood pressure, altered heart rate, and decreased heart contractility (ATSDR 1999). Given that some of these effects occurred at relatively low doses (< 1 mg/kg/day), this appears to be a sensitive target for MeHg’s effects. Human overdose with organic or inorganic mercury has also produced a variety of adverse effects on the heart and blood pressure, and occupational exposure to inorganic mercury has been associated with hypertension and nonischemic heart disease (ATSDR 1999; Boffetta et al. 2001).

Epidemiologic evidence is generally supportive of an association between MeHg body burden in the general public, primarily from fish consumption, and cardiovascular disease (Stern 2005). This database is not as robust as that supporting the benefits of fish oils on CHD, but nevertheless includes substantive findings that need to be accounted for in a risk/benefit analysis. A prospective study of 1,014 Finnish men found that those in the highest quintile of MeHg exposure (hair mercury > 2.81 ppm) had an accelerated thickening of the carotid artery, an indication of atherosclerosis (Salonen et al. 2000). Several studies provide evidence of increased CHD mortality in men in relation to hair or toenail mercury (Guallar et al. 2002; Rissanen et al. 2000; Salonen et al. 1995; Virtanen et al. 2005). In a case–control study spanning eight European countries and Israel, 684 men with MI were found to have significantly greater toenail mercury than the 724 matched controls (Guallar et al. 2002). This association demonstrated a linear dose response that was strengthened when the positive influence of the omega-3 FA DHA was controlled for in the model. An earlier study of 1,833 Finnish men followed prospectively showed a doubling of risk for MI in the highest tertile of exposure (hair mercury > 2 ppm) (Salonen et al. 1995). A follow-up of this eastern Finland population continued to show a heightened risk of coronary events due to MeHg that was able to offset the positive influence of omega-3 FA (Virtanen et al. 2005).

However, several other studies failed to find a consistent association between mercury body burden and cardiovascular outcomes (Ahlqwist et al. 1999; Hallgren et al. 2001; Yoshizawa et al. 2002). A study of 1,462 Swedish women did not find an association between serum mercury and MI or stroke, but that study focused primarily on mercury exposure via amalgam fillings (Ahlqwist et al. 1999). This appears to have been a significant source based on the strong correlations between serum mercury and number of fillings. There was no assessment of fish ingestion or attempt to factor out the benefit of fish oils on the end points measured. In another Swedish study, involving 78 men and women with MI and 124 controls, red blood cell mercury and plasma EPA + DHA were both found to be negative predictors of MI risk (Hallgren et al. 2001). However, the mercury body burden in this population was much lower than in the Finnish studies, possibly too low to have an adverse effect on its own and thus more likely served as a marker for omega-3 FA ingestion from fish. Interestingly, the subgroup with the highest red blood cell mercury and lowest omega-3 FA levels had an elevated odds ratio, but this was not statistically significant possibly due to the low number (10) in this group. Overall, this study did not have sufficient power to detect an independent effect of MeHg on MI, especially given the low exposures to MeHg in this population. Finally, a large prospective study of U.S. health professionals collected toenail mercury data from 33,737 men, of whom 470 had an MI during the course of follow-up (Yoshizawa et al. 2002). The overall analysis showed no difference in risk of MI across the quintiles of toenail mercury, but also in contrast to other studies, there was no demonstrable benefit from fish ingestion. Most subjects were dentists, and they were overrepresented in the highest exposure groups (40% in the lowest quintile; 84% in the upper quintile). The authors reported a positive but nonsignificant association of mercury with CHD in a sub analysis that excluded dentists. This may indicate that MeHg from fish ingestion has a greater influence on cardiovascular risk than inorganic mercury from dental amalgams. Although speculative, this would help explain the negative findings in the Swedish women’s study described above (Ahlqwist et al. 1999).

Overall, mechanistic evidence and results of animal toxicology, human clinical toxicology, and epidemiology studies support the notion that MeHg can be a risk factor for cardiovascular disease. The strongest epidemiology study in this regard is that of Guallar et al. (2002), which provided separate dose–response functions for MeHg risk and omega-3 FA benefit for the same cardio vascular end point. Therefore, we used this study as one of the core studies for our risk/benefit analysis for cardiovascular end points in men.

## Fish and omega-3 FA effects on neurodevelopment

Fish oils, and in particular DHA, have been associated with a number of beneficial effects on neurocognitive and ocular function, both in early life and in old age. These associations include increased visual acuity in newborns (Uauy et al. 2003), better scores on neurodevelopmental test batteries (Daniels et al. 2004; Fleith and Clandinin 2005; Oken et al. 2005, 2008), and prevention of a number of neuropsychiatric disorders in adults, including attention deficit disorder, Alzheimer disease, schizophrenia, and depression (Calon and Cole 2007; Young and Conquer 2005). Dietary supplementation with DHA prevented a number of biochemical changes induced by 1-methyl-4-phenyl-1,2,3,6-tetrahydropyridine (MPTP) in a mouse model of Parkinson disease (Bousquet et al. 2008). The early-life evidence comes from studies in both preterm and full-term infants, with the benefits more consistently shown in preterm infants. These trials have involved the addition of omega-3 FA to infant formula. Part of the impetus for the early-life studies is the finding that formula-fed babies have less plasma and red cell DHA than do breast-fed babies, leading to the question of whether formula should be supplemented with omega-3 FA (Fleith and Clandinin 2005). The strongest association in the fish oil supplementation studies has been with the development of vision, particularly within the first year of life. In addition, maternal ingestion of fish has been associated with enhanced neurocognitive development in ongoing prospective studies (Daniels et al. 2004; Hibbeln et al. 2007; Oken et al. 2005, 2008). Other nutrients in fish may contribute to the neurodevelopmental benefit. However, the fact that this benefit is demonstrable with omega-3 FA supplementation alone indicates an important role for this nutrient (Cohen et al. 2005b).

The present analysis focuses on the evidence of a neurodevelopmental benefit from maternal fish and omega-3 FA ingestion during pregnancy and, in particular, on one study that adjusted for the developmental deficits induced by the concomitant ingestion of MeHg in the fish (Oken et al. 2005), from which it is possible to develop independent dose–response relationships for omega-3 FA benefit and MeHg risk on the same neurodevelopmental end point. Dose–response relationships for MeHg and omega-3 FA effects on IQ have also been derived from a synthesis of the relevant literature (Cohen et al. 2005a, 2005b, 2005c). These other analyses are consistent with the MeHg/omega-3 FA dose responses obtained from the Oken et al. (2005) study that we used as the basis for the present analysis.

## MeHg effects on neurodevelopment

As mentioned above, adverse effects of MeHg have been observed in studies of maternal exposure from fish ingestion in relation to postnatal neuro development. Oken et al. (2005) provided a very useful dose response for this effect because they corrected for the benefit of fish oil ingestion. Several large prospective studies also demonstrate an adverse effect of MeHg, although inconsistencies between them led to considerable debate during the 1990s (Davidson et al. 1998; Grandjean et al. 1997; Kjellstrom et al. 1989). The series of reports from the Faroe Islands are consistent with results from New Zealand in showing an adverse effect of MeHg on neurodevelopment, and this has been judged to outweigh the mostly negative findings from the Seychelles Islands (NRC 2000; U.S. EPA 2001). The epidemiology associations are consistent with an extensive literature in rodents and monkeys demonstrating early-life vulnerability to the neurotoxic effects of MeHg (ATSDR 1999).

## Methods for Integrated Risk/Benefit Analysis

We selected studies from the literature described above to support an integrated risk/benefit analysis for adult cardiovascular and *in utero* neurodevelopmental end points on a species-specific basis. Table 1 summarizes the dose–response relationships found for omega-3 FA and MeHg for common end points: cardiovascular disease in adults (CHD mortality or first MI) and neurodevelopment in 6-month-old infants [visual recognition memory (VRM)]. The adult end points are very similar because both are a measure of coronary artery health; the CHD end point includes fatal MI and sudden death (Mozaffarian and Rimm 2006), whereas the first MI is not necessarily fatal (Guallar et al. 2002). The omega-3 FA benefit on this end point was taken directly from the reported slope for change in relative risk per 100 mg/day intake of EPA + DHA (Mozaffarian and Rimm 2006). This dose response was not adjusted for the counterveiling effect of MeHg and so may underestimate the true relationship or suggest a plateau in benefit that is in fact an indication of MeHg toxicity (see above). We estimated the dose response for MeHg effects on MI from Figure 1A of Guallar et al. (2002) based on the relationship between toenail mercury and MI odds ratios. We used the DHA-adjusted slope from Guallar et al. (2002) in the present analysis. Because the odds ratio is often an overestimate of the relative risk and because the omega-3 FA cardiovascular benefit was in terms of improved relative risk (Mozaffarian and Rimm 2006), we converted the Guallar et al. (2002) data to relative risk by the equation provided by Zhang and Yu (1998). This provides a reasonable estimate of relative risk, although a small (15%) relative bias is possible with this method (McNutt et al. 2003).

This dose response for MeHg effects on MI risk has a hair mercury threshold of 0.51 ppm before any adverse effect is evident (Guallar et al. 2002). Although much of the population in that study had mercury levels in this range and below, there was no clear dose–response trend until the body burden rose above this apparent threshold. The appearance of a threshold may be related to measurement error and variability in the baseline population that obscures a mercury effect below that level. If there is a mercury effect on MI at levels < 0.51 ppm in hair, the slope may be different than that seen at higher body burdens. Therefore, our estimate of mercury MI risk includes this threshold but it is a source of uncertainty.

Infant VRM is a common end point for both omega-3 FA and MeHg because these agents had opposite effects in the 135 mother–infant pairs evaluated by Oken et al. (2005). VRM is a test that evaluates an infant’s ability to encode a stimulus (photograph) into memory and recognize a new stimulus as novel and preferential to the old stimulus. This test is predictive of IQ at later developmental stages (Rose and Feldman 1995). The slope for the hair mercury effect on VRM score was taken directly from Table 2 of Oken et al. (2005), who adjusted the slope for the amount of fish ingestion. Oken et al. (2005) derived the relationship between omega-3 FA intake and VRM score from analysis of food survey records and estimation of omega-3 FA content of fish in relation to the VRM score for each individual, with correction for the inverse association with hair mercury (Oken E, personal communication).

We ran the dose–response functions shown in Table 1 in Excel spreadsheets (Microsoft Corporation, Redmond WA) to estimate the effect of one or more fish meals on the outcome measure using the following risk/benefit equations:


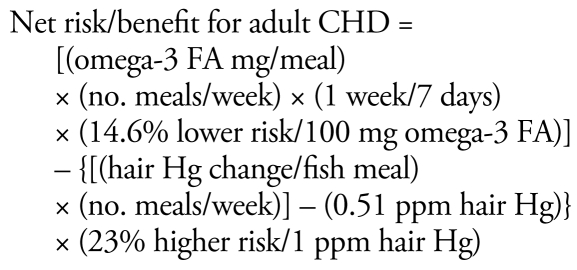



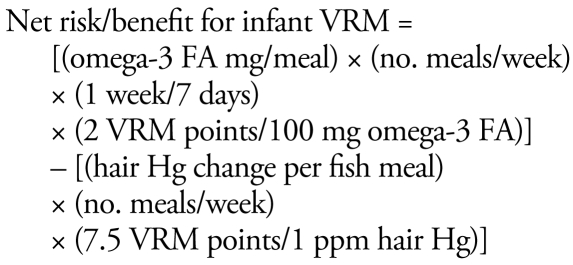


Species that yield a positive result from these equations have a net benefit, whereas a result < 1 signifies an increased risk.

The omega-3 FA CHD benefit may saturate above a certain daily dose, estimated by Mozaffarian and Rimm (2006) at 250 mg/day. However, as described above, this may be an artificial saturation due to the increasing effect of MeHg at higher fish ingestion rates and the evidence of no saturation of benefits in some studies. Therefore, this analysis does not include a saturation function for the omega-3 FA benefit.

These risk/benefit equations contain exposure components based on the number of fish meals eaten per week and the MeHg and omega-3 FA content of the fish. These contents are species specific. Table 2 provides estimates for these fish constituents based on data from the U.S. Department of Agriculture (USDA 2005) for omega-3 FA (DHA + EPA) and from the Food and Drug Administration (FDA 2006) for MeHg. There are a variety of other sources for omega-3 FA content of fish (e.g., American Heart Association 2008; Mozaffarian and Rimm 2006), but these other sources tend to either use the USDA data or to report very similar results. More extensive data for both omega-3 FA and MeHg content of fish (numbers and varieties of fish sampled, seasonal and source variation) are needed to improve confidence and understand variability in this key input data. The list of fish chosen for analysis is based on those commonly available in Connecticut markets and for which MeHg and omega-3 FA data are available. This approach can be applied to any additional species as long as the MeHg and fish oil content of these species are known.

We converted the MeHg fish concentration (micrograms per gram) to a hair MeHg concentration (micrograms per gram) via a one-compartment model that relates MeHg intake to hair mercury as used in the U.S. EPA’s RfD for mercury (Ginsberg and Toal 2000; Rice et al. 2003). The assumed meal size was 6 oz (170 g) of fish, with other parameters used in the model as reported previously (e.g., 95% absorption of MeHg in the gastrointestinal tract; MeHg elimination rate equals 1.4% of body burden per day). We chose a 6-oz meal size to match the recommendation used in the joint FDA/U.S. EPA seafood consumption advisory of two meals per week equivalent to 12 oz of fish (U.S. EPA 2004). Use of the Guallar et al. (2002) dose response required conversion of toenail mercury biomonitoring data to hair mercury. We accomplished this with the factor recently developed by Ohno et al. (2007) (hair mercury in micrograms per gram = 2.44 × toenail mercury in micrograms per gram). They based this factor on the regression slope between hair and toenail mercury in 57 women, which yielded a strong correlation with only a modest degree of variability and few outliers. The strength of the correlation between hair and toenail mercury provides support for the use of toenail mercury as biomarker in the Guallar et al. (2002) study.

## Results

**Figure 1:**
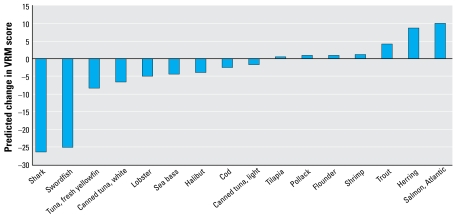
Estimated net effect of MeHg and fish oils on neurodevelopment at 6 months of age, one 6-oz fish meal per week.

**Figure 2:**
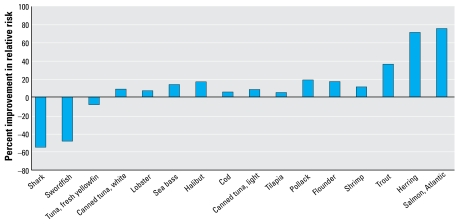
Estimated net effect of MeHg and fish oils on cardiovascular risk, one 6-oz fish meal per week.

**Figure 3:**
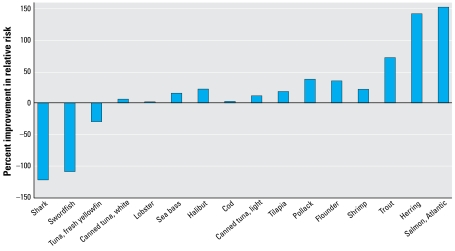
Estimated net effect of MeHg and fish oils on cardiovascular risk, two 6-oz fish meals per week.

Figures 1–3 show the integrated risk/benefit analysis for seafood consumption by end point and species. Figure 1 shows estimates of the influence of MeHg on neuro development at 6 months of age (VRM score) in the 16 fish species chosen for analysis. We modeled these effects based on long-term consumption of one meal per week for several months, enough time to reach steady-state blood and hair concentrations of MeHg. We assumed that the omega-3 FA benefit requires consistent exposure over time and that no other fish were consumed other than the one meal per week of the indicated species.

Figure 1 shows a range of effects, from a large negative effect for swordfish and shark to modest positive effects for trout, farmed salmon, and herring. The rest of the species are in an intermediate zone of rather small net effect in the positive or negative direction. Canned tuna, both light (primarily skipjack) and white (primarily albacore), show negative deflections, with white tuna predicted to have a 3.7-fold larger negative impact than light tuna. Consumption of more than one meal per week on a regular basis would accentuate the pattern shown in Figure 1 because there are no known thresholds or saturation limits to the MeHg decrement or omega-3 FA benefit for neurodevelopment. This means that the intermediate species in the center of Figure 1 would have larger positive and negative deflections the more meals ingested per week. This is a possible concern for species such as fresh tuna (tuna steak), canned white tuna, lobster, and sea bass. In contrast, the marginal benefit of species such as tilapia, pollack, flounder, and shrimp may increase with greater meal frequency. The negative impacts of swordfish and shark and the beneficial effects of trout, farmed salmon, and herring would also be magnified as consumption of these species goes up. However, the presence of other contaminants in species such as farm-raised salmon (Hites et al. 2004) needs to be considered when recommending frequent fish consumption.

Figure 2 shows the net benefit or risk of fish ingestion on CHD mortality and MI. Not surprisingly, the pattern across species is similar to that shown in Figure 1 because, in our framework, the net benefit or risk is contingent upon the ratio of omega-3 FA to MeHg in the fish, which does not change when analyzing different end points. However, the results in Figure 2 suggest that the risk/benefit ratio is more in the benefit direction for CHD mortality compared with VRM score. This can be seen by the number of species with positive deflections in Figure 2 (13) compared with Figure 1 (7), with such commonly eaten foods as canned tuna and cod having a beneficial influence on the cardio vascular end point but negative influence on the neurodevelopmental end point. One reason for the greater benefit of fish consumption on this end point is the MeHg threshold built into this equation. Species in the central portion of Figure 1 have low to intermediate levels of both MeHg and omega-3 FA; these species are at or below the MeHg effect threshold, thus allowing their modest level of omega-3 FA to be the primary influence. The underlying slope factors are also more favorable for a net benefit in the case of cardiovascular risk. However, we estimated a substantial risk for those whose fish ingestion consists of swordfish or shark; the negative deflection reflects an approximately 50% worsening of the relative risk for MI. In contrast, we estimated an approximately 75% improvement for salmon and herring. These effects are magnified for species on either end of the spectrum when simulating two 6-oz meals per week for each species (Figure 3). Intermediate species show little change at two meals per week, an indication that they have surpassed the MeHg toxicity threshold with this increase in consumption, and this prevents a further benefit from more omega-3 FA intake. The species-specific risk/benefit pattern did not change when evaluating four meals per week (data not shown).

The present risk/benefit analysis allows us to tentatively classify these species into categories of fish consumption. Table 3 presents four consumption categories to illustrate how this analytical framework can be used to guide advisories. The species-specific risk/benefit rankings were sufficiently different across end points to yield slightly different advice for those in the neurodevelopmental risk group versus the cardiovascular risk group.

We have tentatively created an unlimited category because, for the end points and constituents analyzed, increasing consumption of certain fish was associated with an increasing benefit. A caveat is the evidence for a saturation of the omega-3 FA cardio vascular benefit > 250 mg/day (Mozaffarian and Rimm 2006), but as described above, saturation of the benefit is speculative for cardiovascular risk and is not evident in the limited analyses available for neurodevelopmental risk (Hibbeln et al. 2007; Oken et al. 2005, 2008). However, because of persistent organochlorine contaminants in certain species such as farmed salmon, one must consider consumption limits based on cancer risk or other end points (Foran et al. 2005). Data for such contaminants should be analyzed to make sure that unlimited consumption of these species is appropriate.

Only a few species are in the twice-per-week consumption category, which is the general seafood advice from the FDA (2004). This is because the largest category is unlimited consumption, containing seven species for the neurodevelopmental risk group. Unlimited consumption is taken to mean one 6-oz meal per day. Figures 1–3 show these species to be associated with net beneficial effects, regardless of the number of meals per week. Fish were placed in this category if they have a net beneficial effect and also if the RfD for mercury (0.1 μg/kg/day) is not exceeded from daily fish ingestion. For the neurodevelopmental risk group, cod and canned light tuna have a slight negative deflection in Figure 1, but were placed in the twice weekly category because when eaten at this frequency they provide less MeHg than the neurodevelopmental RfD and are unlikely to be a significant risk, given the various uncertainties and the fact that there are other nutrients in fish. We included five species in the once weekly category (canned white tuna, tuna steak, halibut, sea bass, lobster). Although we estimated them to yield a net risk at one meal per week (Figure 1), they are also at or below the neurodevelopmental RfD at this frequency. Swordfish and shark have considerably more MeHg, and are estimated to have a much greater net risk and so are in the “do not eat” category. Of the 16 species analyzed, none fit into a one meal per month category, although that may be appropriate for other fish.

For the cardiovascular risk group, unlimited consumption appears to be appropriate for nine species, and potentially several more (Figures 2, 3). However, we downgraded canned white tuna, halibut, sea bass, and lobster to two meals per month because of concerns for neurologic effects. There is no MeHg RfD relevant for the general population, but a number of states have used a 3-fold higher target dose (0.3 μg/kg/day) given the likely differences in sensitivity for neurologic effects between early life and adults (McCann 2005); this target dose is the same as the IRIS RfD for inorganic mercury salts (U.S. EPA 1995). Thus, we placed species in the twice weekly category to keep MeHg exposure below the target dose for the general public to prevent neurologic effects. Tuna steak was placed in the once weekly category to limit the risk as estimated in Figures 2 and 3, which is very small at once per week. We estimated sword-fish and shark to have a substantial net risk, even at one meal per month; thus, they are in the “do not eat” category.

## Discussion

This analysis presents a first attempt at a model that can be refined in the future as more data become available on cardiovascular and neurodevelopmental risks of MeHg, and the health benefits of consuming fish and fish oils. Although we acknowledge that there are limitations in the data used to derive this model, there appears to be an obvious utility to this approach. Public health officials need to weigh the positive and negative aspects of particular fish species when crafting advisories, but to date, there is no well-accepted, objective method to do this. Using this model, we have placed species commonly available in Connecticut into four consumption categories to illustrate the potential utility of the model. These consumption rates can be used as a point of comparison with rates being recommended by the FDA, the U.S. EPA, and various medical and public health authorities, after recognizing the limitations of the present analysis.

We considered the influence of fish consumption on end points that are among the most sensitive for the beneficial effects of omega-3 FA and the toxicity of MeHg. The analysis addresses two completely different groups (adults and the fetus) and encompasses 16 different species, yet it is simplistic in only assessing two factors regarding fish ingestion that may influence these end points. Other nutrients such as protein, selenium, iron, and iodide and other contaminants such as polychlorinated biphenyls, persistent pesticides, and dioxins (Bocio et al. 2007; Hites et al. 2004) may also be contained in these species. We chose constituents (omega-3 FA, MeHg) that have a mechanistic basis for influencing cardiovascular and neurodevelopmental outcomes and have actually been shown to do so in a variety of animal and human studies (Cohen et al. 2005a; IOM 2006; Mozaffarian and Rimm 2006). However, the potential importance of other constituents and end points creates uncertainty regarding the overall health implications of fish consumption.

It is important to recognize that fish ingestion has shown a beneficial effect on neuro developmental and cardiac outcomes in a number of studies (Daniels et al. 2004; Hibbeln et al. 2007; IOM 2006; Mozaffarian and Rimm 2006; Oken et al. 2005). Therefore, an important public health message is that fish are a key dietary component. However, this can also be incorrectly interpreted to mean that, despite MeHg contamination, fish ingestion is a positive influence and consumption limits are unnecessary (Hibbeln et al. 2007). Results from general population studies are likely a reflection of the types of fish eaten. If the studied population ingested more beneficial fish (Figures 1–3), this can create the appearance of a generalizable positive association in the absence of information on the actual species consumed. However, the present analysis and those of others (Guallar et al. 2002; Mahaffey et al. 2007; Oken et al. 2005, 2008; Stern 2007) point out the importance of looking at individual species because the risk/benefit ratio may vary considerably across species. A species-by-species approach to consumption advisories is particularly meaningful given that many people have favorite fish they eat most often. The goal of the species-specific approach is to encourage people to eat from a variety of fish, focusing on the most beneficial species.

Our analysis is supportive of the federal advisory (FDA 2004; U.S. EPA 2004) in showing that certain species should not be eaten by women of childbearing age (swordfish, shark; federal advisory also lists king mackerel and tilefish). In addition, we provide risk/benefit support for separate two meal and one meal per week categories. The federal advisory generally recommends two 6-oz seafood meals per week but does specifically limit canned white tuna to one meal per week. The present analysis goes beyond that to list other species that are candidates for the once weekly category. Further, we provide a list of species that may potentially be safely consumed at greater than the meal frequency recommended by the federal advisory, on the basis of neurodevelopmental and cardio vascular risks, without taking into account other contaminants and end points of potential concern.

This assignment of consumption advice for individual species is tentative given the limitations inherent in the present analysis. The dose–response relationships for the risks and benefits of these components (Table 1) are supported by the available data but do contain uncertainties. The omega-3 FA benefit for acute cardiovascular risk has been documented in numerous epidemiology studies, and the dose response shown in Table 1 is a synthesis of 20 different studies (Mozaffarian and Rimm 2006). However, many of these studies involved fish consumption rather than omega-3 FA supplementation; therefore, the effect of a single nutrient (omega-3 FA) is uncertain, given that other nutrients in fish may have contributed to the observed benefit. Although this remains an uncertainty, omega-3 FA is a well-established benefit for cardiovascular risk and is the main fish nutrient for which dose–response relationships have been reported. Therefore, this is the most feasible approach at the current time. This is not an area of possible underestimation of fish benefits. The benefits attributed to omega-3 FA in the fish consumption studies come from all nutrients, not just the fish oils, because we made no attempt to separate out these other benefits. From this perspective, the omega-3 FA dose–response functions developed in the present analysis will tend to capture the overall benefit of fish consumption, except for the limited extent to which studies of fish oil supplements contribute to the supporting database.

MeHg effects on heart function and blood vessels have been reported in animal studies, cell cultures, and two large epidemiology studies (ATSDR 1999; Guallar et al. 2002; Mazerik et al. 2007; Rissanen et al. 2000; Salonen et al. 1995, 2000; Virtanen et al. 2005). This includes a series of four reports from a group of men in eastern Finland whose diet was enriched in fish that are low in omega-3 FA and relatively high in MeHg (Guallar et al. 2002; Rissanen et al. 2000; Salonen et al. 1995; Virtanen et al. 2005). This cohort provides a good opportunity to document an MeHg effect without much compensation by dietary omega-3 FA. Guallar et al. (2002) studied a different population of men from across Europe and Israel in whom MeHg exposure varied substantially based on country of residence and sources of fish intake. The association of MeHg with increasing cardiovascular risk was evident even without correction for DHA exposure, but the association was strengthened once DHA was considered. These findings provided a reasonable dose response for the present study (Table 1) despite the fact that several studies have not shown such an association with inorganic mercury or MeHg (Ahlqwist et al. 1999; Hallgren et al. 2001; Yoshizawa et al. 2002). One of these was primarily a study of occupational exposure to elemental mercury from dental amalgam (Yoshizawa et al. 2002), another was in Swedish women rather than men and also appears to have amalgam as a primary source of mercury (Ahlqwist et al. 1999), and the more recent Swedish study had too few subjects with elevated mercury exposure (Hallgren et al. 2001). Therefore, these studies are not substantial counterweights to the positive findings in European men described above. However, these positive findings are limited in coming from only two data sets, eastern Finland and the Guallar et al. (2002) results, with a useful dose–response analysis available only in the latter case. It is possible that reanalysis of the eastern Finland data could further support this dose response, because Salonen et al. (1995) found an odds ratio (2.0) for elevated hair mercury (≥ 2 ppm) similar to that found by Guallar et al. (2002). Further exploration of MeHg effects on cardiovascular risk is critical for establishing fish consumption advice that is adequately protective for this end point.

**Figure 4:**
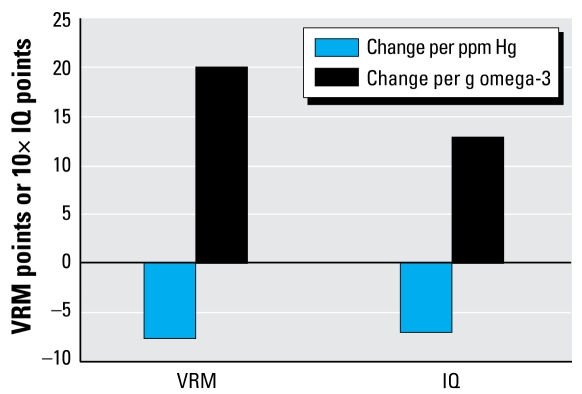
Comparison of estimated effect sizes for MeHg and omega-3 FAs on IQ (Cohen et al. 2005a) and VRM (Oken et al. 2005). Scale for IQ points is multiplied by 10 to adjust size of bars for easy viewing relative to VRM score.

Regarding the neurodevelopmental dose response shown in Table 1, both the omega-3 FA benefit and MeHg risk factors were derived by Oken et al. (2005) from an analysis of VRM scores in 6-month-old children. The group in that study which most clearly showed the MeHg effect was small (high hair mercury, low fish intake; *n* = 12). However, other data corroborate this dose response. Figure 4 shows our comparison of data from Oken et al. (2005) with dose–response factors for a related end point, IQ, as synthesized from several studies (Cohen et al. 2005a, 2005b, 2005c). The ratio of omega-3 FA benefits to MeHg risks is similar across these studies, with the dose response for IQ somewhat less in the benefit direction than the one we used based on VRM. Therefore, it is unlikely that we are under estimating the net fish benefit on neurodevelopment by using the Oken et al. (2005) analysis as our basis. A recent follow-up with this group of mother–child pairs found neurodevelopmental evidence of fish ingestion benefits and MeHg risks extending out to 3 years of age (Oken et al. 2008). There was evidence of a beneficial influence of omega-3 FAs on these outcomes, but this did not attain statistical significance, possibly due to the uncertainties in calculating omega-3 FA intake from diaries of fish consumption. The role of fish oils and other fish nutrients in assisting brain development needs to be a continuing research focus.

Our analysis is limited in that we assessed each fish species in isolation from consumption of any other fish. People generally eat a variety of fish, although some may have a strong preference for one particular species. A robust analysis of food dietary patterns can be used to assess what fish the U.S. population eats (Carrington and Bolger 2002) and how this influences the risk/benefit equation across average or upper-bound consumers. Other variabilities not expressed in our analysis are important to explore and build into more refined analyses: the variability in fish concentrations in omega-3 FA and MeHg, the variability in the toxico kinetics of MeHg, and the variability in response functions for omega-3 FA and MeHg. Although the present analysis does not address population risk, it provides a useful framework for analyzing species-specific risks and benefits that need to be considered when deriving fish advisories. This is critical because a number of states, including Connecticut, are evaluating ways to highlight beneficial fish and discourage consumption of the riskier species.

Several other analyses of the risks and benefits of fish consumption have been published that range from purely qualitative to more quantitative estimates of net risk or benefit. The IOM (2006) provided a qualitative summary and recommended that fish be included in the diet but within federal consumption guidelines. Adults at risk for cardiovascular disease are recommended to eat two 3-oz meals per week as a preventative measure. Above this consumption rate, the IOM recommends diversifying the fish intake to minimize the chance of excessive MeHg exposure from particular fish sources. Mozaffarian and Rimm (2006) performed a more quantitative assessment of dose–response relationships for fish oil benefits on cardiovascular outcomes, but they did not provide a quantitative assessment of MeHg risks. Their risk/benefit assessment was mostly qualitative and concluded that consuming one to two servings of fish per week is beneficial in adults and in women of childbearing age, although the latter group should be wary of a few high MeHg species. In an analysis of seafood available in New Jersey markets, Burger et al. (2005) focused on cost and health considerations but not the benefits of omega-3 FA. They found that flounder was the most economical species that is low in MeHg. Cohen et al. (2005a) used quality-adjusted life years (QALYs) to put the MeHg risks to cognitive development on a common scale with fish benefits for CHD mortality, stroke prevention, and DHA benefits for neuro development. Their analysis looked at how consumption patterns may shift in response to fish advisories and found a net benefit if advisories are properly followed but substantial health risks if advisories lead to unnecessary decreases in fish consumption. Ponce et al. (2000) also used QALYs to contrast MeHg neurodevelopmental risk with fish oil cardiovascular benefits. Their article had the drawback of mixing different end points and receptor types (early life and adult) into a single analysis. Domingo et al. (2007a, 2007b) provided data on omega-3 FA and contaminant levels in 14 species sampled from fish markets in Catalonia, Spain. They analyzed whether certain dietary patterns would result in contaminant intakes above tolerable daily intakes and whether omega-3 FA intakes were adequate with respect to recommendations of international heart associations. In a somewhat similar vein, Foran et al. (2005) quantitatively assessed the risks and benefits of farmed and wild salmon consumption with benefits entered into the equation as the omega-3 FA content of the meal and the risk assessed based on the cumulative cancer or noncancer risk of the contaminants (target cancer risk, 1 in 10^−5^, target noncancer risk of unity). Their analysis found that farmed salmon should be limited, from less than one meal per week to three meals per week depending on source, to meet the World Health Organization (1998) target dose for dioxin equivalents (1 pg/kg/day), with farmed salmon from European sources generally on the low end of this consumption advice. These frequencies were still associated with elevated cancer risk, although they also contained substantial omega-3 FA benefit. It is important to keep in mind that trace levels of carcinogens are in many foods, so the relative risk/benefit ratio of a source such as salmon should ideally be compared against other protein sources (e.g., meat, dairy, vegetarian sources) if cancer is a critical end point. Along these lines, the levels of dioxins found in some farmed salmon are greater than what is typically available from other protein sources. The Connecticut Department of Public Health’s latest seafood advisory is for no more than one meal per week of farmed salmon on this basis.

It may be theoretically possible to obtain omega-3 FA benefits and avoid some of the contaminant issues by taking fish oil supplements. Other foods that are fortified with omega-3 FA, such as eggs and milk, can be an additional source. For example, chickens fed diets containing ground flaxseed lay eggs that are enriched in omega-3 FA (~ 500 mg/egg), although most of this is in the form of α-linolenic acid, which has less evidence for neuro developmental and cardiovascular benefits compared with fish oils (FDA 2005). Another form of omega-3 FA fortification of eggs has been developed that involves supplementation of the hen’s diet with marine microalgae, a source reportedly rich in DHA (150 mg/egg). Publicly available test data regarding the omega-3 FA content of these supplemented foods are needed to understand their potential benefit. Another consideration is that replacement of fish with supplements or fortified eggs will not necessarily provide other nutrients that fish offer (e.g., iron, selenium, iodide). These nutrients are not being analyzed in present risk/benefit analyses but they may be part of the benefit being attributed to fish-oil ingestion. In general, nutrition authorities recommend obtaining nutrients from the whole food rather than from extracted or chemically synthesized components. Finally, omega-3 FA supplements are not regulated by the FDA, so label accuracy, quality control, and contaminant testing may be issues. Clearly, the beneficial effects of omega-3 FAs on cardiovascular and neurodevelopmental outcomes need to be further explored in relation to the overall benefits of fish consumption to refine species-specific advice and to make recommendations about the utility of fish oil supplements.

In contrast to previous risk/benefit analyses, the present study is the first to provide an integrated analysis for MeHg and omega-3 FA that uses dose–response relationships on common end points and that evaluates the net effect on a species-by-species basis. This approach and the resulting consumption categories illustrate a framework that should be helpful in establishing advisories for a wide variety of commercially available and locally caught fish, assuming that the requisite MeHg and omega-3 FA data are available. We believe this can help resolve the confusion that currently exists regarding fish consumption and yield a message that focuses on the most beneficial fish choices without eliciting fear over the dangers of MeHg. Currently, there are numerous uncertainties regarding additional contaminants, nutrients, end points, underlying dose–response functions, and comparisons with other protein sources. These factors would require a more data intensive and complex analysis, but this is an important direction for the future (Domingo et al. 2007b; Foran et al. 2005).

## Figures and Tables

**Table 1:** Dose–response relationships for key MeHg and omega-3 FA end points.

End point	Agent	Dose response	Comments	References
Adult CHD mortality	Omega-3 FA	14.6% decreased relative risk per 100 mg/day	Combined data across 20 studies for EPA + DHA intake versus CHD mortality; possible saturation of benefit > 250 mg/day	Mozaffarian and Rimm 2006
Adult MI risk	MeHg	23% increased relative risk per 1 ppm hair Hg	Slope adjusted for DHA content of lipid as index of fish oil intake; risk not apparent < 0.51 ppm hair Hg; toenail Hg measured but converted to ppm in hair	Toenail to hair Hg conversion, Guallar et al. 2002, Ohno et al. 2007; odds ratio conversion to relative risk, Zhang and Yu 1998
Infant VRM score	Omega-3 FA	2.0-point increase per 100 mg/day	VRM measured at 6 months in 135 mother–infant pairs; fish oil intake estimated from dietary survey	Oken et al. 2005
Infant VRM score	MeHg	7.5-point decrease per 1 ppm hair Hg	VRM measured at 6 months in 135 mother–infant pairs; direct measurement of maternal hair Hg	Oken et al. 2005

**Table 2:** Estimated omega-3 FA and MeHg levels in commonly eaten fish.

Fish species	Omega-3^a^ (mg/6 oz)	MeHg^b^ (μg/g)
Cod, Atlantic	269	0.11
Flounder/sole	852	0.05
Halibut	1,398	0.26
Herring, Atlantic	3,424	0.04
Lobster	1,129	0.24
Pollack	922	0.06
Salmon, Atlantic, farmed	3,658	0.014
Sea bass	1,295	0.27
Shark	1,170	0.99
Shrimp	536	0.01
Swordfish	1,392	0.97
Tilapia	240	0.01
Trout	1,744	0.03
Tuna, canned, light	425	0.12
Tuna, canned, white	1,462	0.35
Tuna, fresh, yellowfin	474	0.325

a Omega-3 FA represents the sum of EPA and DHA. Shark data from Mozaffarian and Rimm (2006); other data from USDA (2005),

b MeHg data from FDA (2006); data for salmon reported as fresh/frozen and not distinguished according to source.

**Table 3:** Tentative fish consumption categories for the 16 species analyzed in the present risk/benefit assessment (based on 6-oz meal size).

Risk group	Consumption category	Fish species
Neurodevelopmental^a^	Unlimited (pending evaluation of other contaminants)^b^	Tilapia, pollack, flounder, shrimp, trout, herring, salmon
	Twice per week	Canned light tuna, cod
	Once per week	Canned white tuna, tuna steak, halibut, sea bass, lobster
	Do not eat	Swordfish, shark
Cardiovascular^c^	Unlimited (pending other contaminants)^c^	Tilapia, pollack, flounder, shrimp, trout, herring, salmon, canned light tuna, cod
	Twice per week	Canned white tuna, halibut, sea bass, lobster
	Once per week	Tuna steak
	Do not eat	Swordfish, shark

a Pregnant women, women of childbearing age, nursing mothers, young children.

b Unlimited taken to mean daily consumption.

c General adult population.
